# Lipid Metabolism in Macrophages: Focus on Atherosclerosis

**DOI:** 10.3390/biomedicines8080262

**Published:** 2020-08-01

**Authors:** Vasily N. Sukhorukov, Victoria A. Khotina, Yegor S. Chegodaev, Ekaterina Ivanova, Igor A. Sobenin, Alexander N. Orekhov

**Affiliations:** 1 Research Institute of Human Morphology, Laboratory of Cellular and Molecular Pathology of Cardiovascular System, 3 Tsyurupy Str., 117418 Moscow, Russia; nafany905@gmail.com (V.A.K.); igor.sobenin@gmail.com (I.A.S.); a.h.opexob@gmail.com (A.N.O.); 2 Russian Medical Research Center of Cardiology, Institute of Experimental Cardiology, Laboratory of Medical Genetics, 15-a 3-rd Cherepkovskaya Str., 121552 Moscow, Russia; 3 Sechenov First Moscow State Medical University, 119146 Moscow, Russia; egozavr-ch@mail.ru; 4 Institute for Atherosclerosis Research, Skolkovo Innovative Center, 121609 Moscow, Russia; kate.ivanov@gmail.com; 5 Institute of General Pathology and Pathophysiology, Laboratory of Angiopathology, 8 Baltiyskaya Str., 125315 Moscow, Russia

**Keywords:** lipid metabolism, foam cell, macrophage, atherosclerosis, lipid droplet

## Abstract

Mechanisms of lipid homeostasis and its impairment are of crucial importance for atherogenesis, and their understanding is necessary for successful development of new therapeutic approaches. In the arterial wall, macrophages play a prominent role in intracellular lipid accumulation, giving rise to foam cells that populate growing atherosclerotic plaques. Under normal conditions, macrophages are able to process substantial amounts of lipids and cholesterol without critical overload of the catabolic processes. However, in atherosclerosis, these pathways become inefficient, leading to imbalance in cholesterol and lipid metabolism and disruption of cellular functions. In this review, we summarize the existing knowledge on the involvement of macrophage lipid metabolism in atherosclerosis development, including both the results of recent studies and classical concepts, and provide a detailed description of these processes from the moment of lipid uptake with lipoproteins to cholesterol efflux.

## 1. Introduction

Intracellular lipid accumulation associated with atherosclerosis can affect virtually any of the cell types that can be found in the subendothelial space of human arteries, including vascular smooth muscular cells (VSMCs), stellate pericyte-like cells, and macrophages. Although macrophages represent only a minority of the cellular population of the subendothelial space, they play an important role in triggering atherosclerosis through active lipid accumulation and stimulation of proinflammatory response. Macrophages are recognized as a major precursor of foam cells. These cells have their cytoplasm filled with lipid droplets, which gives them their specific microscopic appearance. Such cells are typically present in growing atherosclerotic plaques. Proatherogenic modified low-density lipoprotein (mLDL) is the main source of cholesterol and lipids accumulating in the arterial wall, which plays a key role in atherosclerosis [1]. The balance of lipid biosynthesis and degradation in the cells is important for maintaining cell structure and functioning. Lipid homeostasis is regulated at several levels, including receptor-mediated uptake, synthesis, storage, metabolism, and efflux. Disturbances of cellular lipid metabolism can contribute to the development of certain human diseases, in particular, atherosclerosis [2].

Macrophages are considered to be the main cellular participants in atherogenesis in the intima of the blood vessels. Under normal conditions, LDL entering the cell through receptor-mediated endocytosis can be degraded in the lysosomes, which prevents excessive lipid accumulation. However, in atherosclerosis, this process is disturbed. It was shown that mLDL may be internalized not via the normal route mediated by low-density lipoprotein receptor (LDLR), but via phagocytosis mediated by less specific scavenger receptors that are expressed on macrophages and other phagocytic cells. Therefore, atherogenic lipoprotein particles provoke intracellular lipid accumulation and foam cell formation in the intima of vessels. Under normal physiological conditions, a tightly regulated balance of uptake, synthesis, and efflux, is maintained in the cells. In case of free cholesterol excess, which is detected by cellular regulatory mechanisms described below, the amount of LDLR on the cell surface decreases, reducing the uptake of exogenous lipids with native LDL. However, presence of atherogenic mLDL causes an imbalance of these processes due to the unregulated uptake of mLDL by macrophages, which ultimately leads to excessive accumulation of lipids by the cell and foam cell formation. The process of excessive lipid accumulation inside the cell is based on the mechanisms of uptake, trafficking, autophagy, metabolic cycles, accumulation, and neutralization of free cholesterol and fatty acids (Table 1) [3].

## 2. LDL Uptake: Role of Receptors

Macrophages may internalize native LDL, as well as mLDL, through different pathways. Known LDL modifications, occurring in vivo, include desialylation, oxidation, and formation of LDL self-associates and LDL-containing immune complexes, which is facilitated by certain physicochemical modifications of the LDL particle [4].

Almost all receptors recognizing LDL, both native and modified, belong to different classes of scavenger receptors. To date, eight classes of scavenger receptors (classes A–H) have been identified. The receptor to native LDL, LDLR, belongs to class L of scavenger receptors. This class also comprises LDLR-related protein 1 (LRP1) and VLDLR (very-low-density lipoprotein receptor). Receptors from classes A, B, D, E, and F can bind mLDL [5]. Under normal physiological conditions, LDLR expressed on macrophages mediates the endocytosis of native LDL that does not lead to lipid accumulation and subsequent foam cell formation [6]. The indirect interaction of LDLR with adaptor protein complex 2 (AP-2) through the adaptor protein ARH is necessary for internalization of LDL–LDLR complex via clathrin-coated vesicles. Clathrin-coated vesicles turn into the early endosomes, where dissociation of LDL from the receptor occurs, and then into the late endosomes, from where LDLR can be recycled back to the cell surface or degraded in the lysosomes. In turn, late endosomes fuse with lysosomes, which results in LDL enzymatic degradation to amino acids, fatty acids, and cholesterol. The fate of LDLR depends on the level of free cholesterol in the cell and is regulated by several proteins. In case of low cholesterol concentration, LDLR recycles back to the surface as a complex with COMMD/CCDC22/CCDC93 (CCC) and the Wiskott–Aldrich syndrome protein and SCAR homologue (WASH) [6]. Alternatively, the receptor binds with proprotein convertase subtilisin/kexin type 9 (PCSK9), which promotes LDLR lysosomal degradation and thereby reduces the abundance of this receptor on the cell surface under high free cholesterol concentrations [7]. The expression of LDLR is regulated by Liver X receptor (LXR), which acts as cellular free cholesterol concentration sensor and mediates the expression of sterol regulatory element binding protein-1c (SREBP-1c) [8]. An important regulator of LDLR, PCSK9, and SREBP-1c expression is SREBP-2. In addition, SREBP-2 is necessary to produce an LXR ligand required for normal SREBP-1c expression [9].

Under pathological conditions, native LDL uptake by macrophages may, however, lead to cholesterol accumulation and foam cell formation through receptor-independent, fluid-phase pinocytosis [10]. Pinocytosis may be regulated by phosphoinositide 3-kinase (PI3K), LXRs, the macrophage colony-stimulating factor (M-CSF) receptor, and protein kinase C (PKC) [11].

Oxidative modification of LDL and LDL binding with proteoglycans of the extracellular matrix in the intima of blood vessels lead to aggregation of lipoprotein particles. Aggregated modified LDL (agLDL) may cause lipid accumulation in macrophages in two ways. First, it is known that agLDL is recognized by LRP1 and engulfed via phagocytosis [12,13]. Second, as it was shown recently, macrophages may provide degradation of agLDL via lysosomal synapses, which are extracellular hydrolytic compartments [14]. Toll-like receptor 4 (TLR4) and its adaptor protein MyD88 (myeloid differentiation primary response 88) play the key role in recognition of agLDL, lysosomal synapse formation, and uptake of LDL catabolism products. Moreover, TLR4 launches spleen tyrosine kinase/phosphoinositide 3-kinase/Akt (SYK/PI3K/Akt) signaling pathway, which facilitates the synapse formation and lipid accumulation in macrophages [14].

According to current understanding, the main contribution to foam cell formation is made by the uptake of modified lipoproteins by various classes of scavenger receptors (SR) such as SR class A (SR-A1, also known as CD204), SR class B (CD36), and the lectin-like oxidized LDL 1 receptor (LOX-1, or SR-E1), which are able to recognize and bind modified LDL [15]. SR-1 class A is subdivided into SR-AI, SR-AII, and SR-AIII. The latter is not involved in mLDL uptake and often acts as a regulator of the first two receptors [16]. The expression of SR-A on the surface of macrophages is regulated by various factors. Sac1 and Sac3 phosphatases localized in the endoplasmic reticulum (ER) may play the key role in maintaining a constant level of SR-A expression [17]. Sac1 can facilitate the transport of cholesterol from the ER to the trans-Golgi network and may positively regulate translational or post-translational modifications of expressed receptor molecules. Upregulation of Sac1 expression leads to an increase of SR-A receptor abundancy. Additionally, proinflammatory cytokines, such as tumor necrosis factor (TNF)-α and interleukin (IL)-6, can also upregulate the expression of SR-A, which can result in accumulation of LDL by macrophages under conditions of chronic inflammation within the vessel wall [18]. However, an intracellular mechanism that reduces the uptake of mLDL by macrophages through SR-A has recently been discovered. This mechanism of serine-threonine phosphatase LKB1 promotes phosphorylation and lysosomal degradation of SR-A, resulting in a decrease of the receptors abundance on the cell surface [19].

Another key scavenger receptor, involved in lipoprotein uptake and lipid metabolism, is CD36, whose ligands are mLDL, HDL, fatty acids, and triacylglycerol-rich lipoprotein particles, such as VLDL [20,21,22]. Interaction between mLDL and CD36 activates signaling pathways mediated by nonreceptor tyrosine kinase (SRC), cJun NH2-terminal kinase (JNK), Rac (GTPase) protein, and nuclear factor-κB (NF-κB transcription factor), which enhance LDL absorption, oxidative processes, and the production of proinflammatory cytokines [23]. Recent studies have shown that one of the regulators of CD36 expression is CD146, which promotes internalization of oxLDL [24].

Another scavenger receptor implicated in atherosclerosis-associated lipid accumulation is lectin-like oxidized LDL-1 receptor (LOX-1), a member of the E class SR family. Although LOX-1 expression is low under normal conditions, the development of inflammation and increased oxidative processes in the intima can increase the receptor expression and subsequent uptake of oxLDL [25]. PCSK9, which affects the expression of LDLR in macrophages, can also upregulate the expression of SR-A, CD36, LOX-1, and the absorption of oxLDL in the presence of TNF-α [26].

In addition, several other mLDL-binding receptors of secondary importance have been identified. CD68 is a member of class D scavenger receptors and plays only a minor role in the uptake of mLDL [27]. SCARF1 (the first class F scavenger receptor) can bind mLDL in endothelial cells [28] and participate in clearance of apoptotic cells by macrophages [29]. SR-PSOX/CXCL16 receptor of class G may bind oxLDL in macrophage-like human THP-1 cells [30]. Finally, scavenger receptors, as well as TLR2, TLR3, TLR4, and TLR9, are combined into the pattern recognition receptor (PRR) group and are involved not only in lipid uptake, but also in the activation of inflammatory cytokine production by macrophages [31].

## 3. Intracellular Lipid Trafficking and Storage

Intracellular cholesterol trafficking starts from the early endosome formation, where LDL particles are transferred into the endosome from the cell plasma membrane. Receptors to LDL are recycled from the early endosomes, when LDL are transferred to the late endosomes that fuse with lysosomes, forming endolysosomes with acidic luminal pH [32]. A lysosome contains up to 60 different hydrolytic enzymes, such as proteases, nucleases, and lipases [33], including lysosomal acid lipase (LAL), which converts cholesterol esters and triglyceride molecules from LDL particles into free cholesterol and fatty acids, respectively [34]. Hydrolyzed fatty acids are substrates for production of VLDL and fatty acid β-oxidation cycle (FAO) [35]. Modified lipids can trigger lysosomal stress and subsequently upregulate the *LIPA* gene expression in macrophages [36]. Moreover, lipid-laden foamy macrophages display a higher level of *LIPA* expression in comparison to nonfoamy ones, which have highly activated proinflammatory genes [37,38,39]. Additionally, there are evidences that modified LDL-induced lysosomal stress leads to impaired lysosomal acidification, which interferes with proper LAL activity [36]. Thus, increased LAL activity in lipid-laden macrophages might be completely inefficient in handling the increased influx of lipids.

Niemann–Pick type C (NPC) 1 and 2 proteins [40] and lysosomal-associated membrane protein (LAMP)1 and 2 are required to transfer cholesterol out of the lysosome [41]. LAMP2 facilitates shuttling of cholesterol from NPC2, which is located in the lysosomal lumen, to NPC1, which is a transmembrane protein located in the lysosomal membrane [41]. Lysobisphosphatidic acid (LBPA) is a specific component of endosomal LBPA-membrane that plays an important role in controlling the endosomal cholesterol trafficking. It was shown that direct interaction of NPC2 with LBPA facilitates the transfer of cholesterol to the membrane and further, out of the lysosome. Moreover, it was also found that LBPA-membranes may control both cholesterol storage capacity of endolysosomes and cholesterol egress [42].

From LBPA-membranes, cholesterol can be transferred to the ER, Golgi apparatus, and plasma membrane via both vesicular and nonvesicular routes of intracellular lipid trafficking [43]. Moreover, it can even be transported to mitochondria [44]. Surprisingly, energy-dependent vesicular transport is less abundant than the nonvesicular way. Thus, most lipid trafficking occurs in the membrane contact sites that connect the ER with the Golgi apparatus, mitochondria, plasma membrane, endosomes, lysosomes, and peroxisomes [45,46,47,48]. Nonvesicular transport is mediated by several classes of lipid-binding proteins, such as oxysterol binding protein-related proteins (ORPs) [49], related VAD1 (vascular associated death 1) analog of star related lipid transfer (VASt), synaptotagmin-like mitochondrial lipid binding protein (SMP) [50], and steroidogenic acute regulatory protein related lipid transfer (START) [51], which separate hydrophobic cholesterol from the aqueous environment of the cytoplasm.

From late endosomes, cholesterol and other lipids are transferred to the ER, which has a key role in cholesterol synthesis, sensing, and redistribution, as well as protein synthesis, folding, and transport within the cell [52,53,54]. The ER membranes maintain a stably low cholesterol concentration, which is necessary for optimal functioning of the ER-related enzymes. SREBP2 and nuclear factor erythroid 2 related factor-1 (Nrf1) are the main factors that help regulate the cholesterol concentration [55].

Nrf1, which is thought to be the core component in cholesterol homeostasis, senses cholesterol by physical interaction in the ER membrane, mediates CD36 and LXR activities, and, together with SREBP2, regulates the cellular cholesterol level [55]. SREBP2 mediates cholesterol synthesis under low cholesterol concentration. Under low and normal cholesterol concentration, Nrf1 is located in the nucleus where it blocks CD36 and LXR gene expression. By contrast, under high cholesterol concentration, Nrf1 is retained in the ER through binding with cholesterol. Inhibition of LXR and CD36 expression by Nrf1 is therefore ceased, and cholesterol efflux and CD36-driven inflammation are increased [55].

If the rate of cholesterol influx becomes too high and mechanisms of cholesterol homeostasis regulation do not work properly, it can lead to ER stress initiation. The main sensors of the ER stress caused by excessive cholesterol are PERK (protein kinase R (PKR)-like endoplasmic reticulum kinase) and IRE1 (inositol-requiring enzyme 1), which, in turn, launch the unfolded protein response (UPR). The UPR is activated mainly in response to accumulation of unfolded proteins in the ER lumen [56]. However, it was recently shown that cholesterol can directly activate IRE1- and PERK-related signaling pathways without any perturbation of protein folding [57]. Thus, PERK and IRE1 sense the cholesterol concentration changes via their transmembrane domains and launch the UPR, which may lead to apoptosis [58]. Other consequences of the UPR are upregulation of scavenger receptors (CD36, SR-A1) and downregulation of ABCA1, ABCG1, and SR-B1, which leads to foam cell formation [59]. UPR signaling is also associated with perturbations in calcium homeostasis in the ER through the inhibition of the SERCA pump in lipid-loaded macrophages [60].

Since free cholesterol is toxic for the cell, it can be detoxified through esterification and stored as cholesterol ester in lipid droplets [61]. Transformation of free cholesterol to cholesteryl esters is catalyzed by acyl-coenzyme A:cholesterol acyltransferases (ACAT). The ACAT proteins comprise ACAT1, ACAT2, and diacylglycerol acyltransferases 1 and 2 (DGAT1 and 2). ACAT1 converts free cholesterol into cholesterol ester [62]. ACAT1 can be regulated by various signaling pathways, some of which enhance its expression (MAP, Jak, Erk kinase cascades), while others decrease it (Jnk, NF-κB) [63].

Fatty acids, generated as a result of LDL degradation, are converted to inert triglycerides and driven to lipid droplets to be stored by cells in this inert form. Alternatively, fatty acids can be transported from the droplets to the mitochondria to produce NADH, FADH_2_, and acetyl-CoA via fatty acid β-oxidation cycle (FAO) and ATP via the tricarboxylic acid (TCA) cycle [64]. Triacylglycerols (triglycerides) are synthesized between the two leaflets of the ER membrane by DGAT1 and DGAT2. DGATs convert diacylglycerol and fatty acyl-CoA to triacylglycerol [65]. By contrast, the sequential action of adipose triglyceride lipase (ATGL), monoacylglycerol lipase (MAGL), and hormone-sensitive lipase (HSL) convert triacylglycerol back to fatty acids [66]. Before the transfer to mitochondria, fatty acids are converted by long chain fatty acyl-CoA synthetase to fatty acyl-CoA. Subsequently, fatty acyl-CoA interacts with carnitine via action of carnitine palmitoyltransferase I (CPTI) and is transported to the mitochondria as fatty acyl carnitine, where FAO takes place [64]. Malonyl-CoA can prevent the transfer of fatty acyl carnitine to mitochondria by inhibition of CPTI [67,68].

The excess amount of free cholesterol and other neutral lipids is transferred from the ER to lipid droplets to be stored there. The lipid droplet is a unique organelle, which consists of the core made of neutral lipids, surrounded by a phospholipid monolayer with integral proteins. The lipid droplet also stores potentially toxic lipids, such as fatty acids, and may protect the cell from lipotoxicity, oxidative and ER stress, and autophagy. Lipid droplets interact and exchange cholesterol and other lipids with the ER, lysosomes, endosomes, and mitochondria [69]. Moreover, lipid droplets are considered as central regulators of lipid uptake, trafficking, metabolism, and signaling in the cell [70].

The mechanism of lipid droplet biogenesis is being thoroughly studied, but remains insufficiently understood to date. The first step of lipid droplet biogenesis is sterol esters synthesis by ACAT1 and ACAT2, and triacylglycerols synthesis by DGAT1 and DGAT2 [65]. Accumulation of neutral lipids in the ER membrane leaflets leads to the formation of the lens structure, which enlarges and buds from the ER membrane, forming a lipid droplet. Lipid droplet budding is promoted by FIT1 and FIT2 (fat storage-inducing transmembrane) [71], the conserved ER membrane protein seipin [72], and perilipins (Pln1) [73]. After budding, lipid droplets enlarge through droplet–droplet fusion, transfer of neutral lipids via ER membrane bridges, or even through triacylglycerol synthesis by lipid droplet-localized triacylglycerol synthesis enzymes [74]. Phospholipids from the ER membrane are also required for growing lipid droplet. Fatty acids stimulate the activity of CTP:phosphocholine cytidylyltransferase-α (CCTα), which participates in phosphatidylcholine synthesis [75]. Moreover, CCTα upregulation induces inflammatory response in adipose tissue macrophages [76]. Lipid droplets can either stay attached to the ER or detached from it [77]. In addition, lipid droplets may play an important role in alleviating ER stress as they can store the excess amounts of fatty acids, remove phospholipids from the ER membrane, and, moreover, might be involved in the clearance of misfolded proteins [78].

In addition to the membrane contact sites with the ER, which are built during biogenesis, lipid droplets can also form contacts with most cellular organelles such as Golgi, mitochondria, lysosomes, nucleus, and peroxisomes [79]. These membranes contact sites facilitate the exchange of lipids, ions, and metabolites [80].

Contact sites between lipid droplets and mitochondria allow release of fatty acids, which are used by mitochondria for energy production via TCA and FAO [3]. In addition to mitochondria, peroxisomes are an important site for β-oxidation of very-long-chain fatty acids and branched fatty acids [81]. Alternatively, enhanced ATP synthesis in mitochondria may serve a fuel for triacylglycerol synthesis. Thus, the mitochondria–droplet interaction may mediate both lipogenesis and lipolysis under different metabolic conditions [69]. Moreover, during autophagy, lipid droplet biogenesis may regulate consumption of acylcarnitine by mitochondria, which prevents mitochondrial dysfunction because of excessive acylcarnitine accumulation [82].

## 4. Lipid Biosynthesis

One of the key enzymes in intracellular cholesterol biosynthesis is HMG-CoA reductase localized in the ER, which catalyzes mevalonate synthesis. This is the limiting stage of cholesterol synthesis in cells. Cholesterol is synthesized from mevalonate by a series of successive reactions. After synthesis, cholesterol leaves the ER and is delivered to the plasma membrane. Inhibition of the HMG-CoA reductase gene in macrophages leads to a decrease in the migration activity of monocytes and macrophages to foci of atherosclerotic lesions [83]. Lipid synthesis involves a series of sequential enzymatic reactions in which triglycerides, fatty acids, and cholesterol are formed from acetyl-CoA [84].

The SREBP family includes three proteins—SREBP1a, SREBP1c, and SREBP2—and activates lipid synthesis under lipid deficiency. SREBP1 controls the transcription of genes, such as the fatty acid synthase (FASN) gene, which are involved in fatty acid biosynthesis. At the same time, SREBP2 regulates cholesterol biosynthesis, intracellular transfer of lipids, and LDL uptake [85]. SREBP forms a complex with SCAP, which contains a sterol-sensing domain that allows detecting fluctuations of cholesterol concentration. The SREBP–SCAP complex is retained in the ER membrane by insulin-induced gene 1 (INSIG1) under normal and high cholesterol concentration. When cholesterol concentration drops below 5%, INSIG1 dissociates from the complex and remains anchored in the membrane, allowing SREBP-SCAP to be transferred to the Golgi apparatus. Once on the Golgi membranes, SREBP matures and is further translocated to the nucleus, where it activates the expression of lipid synthesis genes [85].

When the concentration of intracellular lipids decreases, fatty acids synthesis is activated by SREBP1c and upstream stimulatory factors (USF1 and USF2) in the cytoplasm, thanks to which, cells can synthesize lipids from precursors originating from other metabolic pathways, such as TCA, glycolysis, and pentose phosphate pathways.

**Figure 1:**
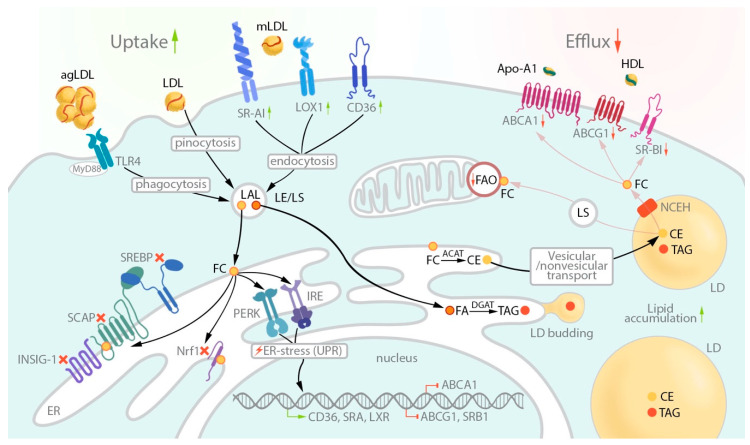
Mechanisms of lipid metabolism leading to lipid-laden macrophages. Black arrows represent the lipid influx and accumulation; grey arrows represent pathways of cholesterol efflux and metabolism. mLDL is bound to several scavenger receptors (SR) of macrophages, such as SR-A1, CD36, and lectin-like oxidized low-density lipoprotein (LDL) receptor-1 (LOX-1). TLR4, together with MyD88, mediates phagocytosis of aggregated LDL, while native LDL may be taken up by cells via pinocytosis. In late endosomes/lysosomes (LE/LS), lysosomal acid lipase (LAL) degrades cholesteryl esters (CE) from LDL particles to free cholesterol (FC) and fatty acids (FA). Then, FA and FC are transferred to the endoplasmic reticulum (ER) membrane, where acyl coenzyme A: cholesterol acyltransferase-1 (ACAT1) converts FC to CE. In the ER, FC interacts with SCAP, arresting cholesterol de novo synthesis, and with Nrf1, which provokes CD36 expression. Triacylglycerols (TAG) are synthesized from FA-CoA and diacylglycerols by DGAT1 in the ER membrane. TAG accumulation in the ER membrane bilayers mediates lipid droplets (LD) growth and budding from the ER. Cholesteryl esters are transported to lipid droplets via vesicular/nonvesicular transport and are stored there. Neutral cholesterol ester hydrolase (NCEH) transforms CE back to FC, which is transported outside the cells via ATP-binding cassette (ABC) transporters A1 and G1 (ABCA1 and ABCG1), as well as via SR-BI. Apolipoprotein A-1 (ApoA-1) is an acceptor for FC carried by ABCA1. HDL takes FC that is transferred via ABCG1 and SR-BI. In the lysosome (LS), TAG from lipid droplets can be transformed by LAL into FA, and FA may be used as fuel for fatty acid oxidation (FAO) in mitochondria to produce energy for a cell. This machinery maintains lipid and cholesterol homeostasis. In atherosclerosis, especially in M1 macrophages, this homeostasis is disturbed. Deregulated uptake of mLDL via SR leads to the high concentration of FC and other lipids in the ER and lipid droplets. In the ER the exceeding amount of free cholesterol activates IRE1 and PERK, which is a sensor of unfolding protein response (UPR) and triggers the ER stress. The UPR leads to upregulation of CD36, SRA, and LXR expression, which enhances mLDL uptake, and to downregulation of ABCA1 and ABCG1 transporters, and SR-BI, which diminishes cholesterol efflux. Moreover, FAO is decreased that promotes TAG accumulation in lipid droplets. Together, these processes lead to exorbitant deposition of lipids in lipid droplets, and, consequently, foam cell formation from lipid-loaded macrophages.

Fatty acid synthesis from acetyl-CoA and malonyl-CoA in the presence of NADPH in macrophages is catalyzed by FASN, which is found to be located both in the cytosol and in the mitochondria [86]. Malonyl-CoA is a key metabolite that regulates fatty acid synthesis, such as FAO. At a low cellular level of malonyl-CoA, fatty acids are involved in triglyceride production, and at high levels of malonyl-CoA, fatty acids are prevented from entering mitochondria and subsequently from FAO [87]. Lipogenesis in macrophages is necessary not only for maintaining the stability of lipid membrane composition, but also for synthesis of inflammatory mediators, in particular, in macrophages that have a proinflammatory (M1) phenotype. FASN deficiency leads to changes of the plasma membrane composition and reduced transmission of proinflammatory signals [64]. Moreover, it was shown that FAO activity correlates with the type of macrophage polarization. Specifically, the alternative (anti-inflammatory) M2 macrophage polarization leads to upregulation of FAO and lipolysis via LAL upon IL-4 stimulation. On the contrary, in M1 macrophages, lipolysis, and FAO are strongly downregulated, while oxLDL uptake, as well as CD36 expression, and lipid droplet biogenesis are increased [3]. As a result, M1 macrophages can accumulate more lipids than M2 macrophages and can serve as a source of foam cells in the atherosclerotic lesion (Figure 1).

In addition, atherosclerosis can be accompanied by mitochondrial DNA (mtDNA) mutations that are associated with proinflammatory signaling and foam cell formation [88]. Mutations in mtDNA can lead to defective function of mitochondrial origin proteins, which participate in energy production and code tRNAs. These mtDNA mutations contribute to impaired TCA and ATP production, oxidative stress and promotion of atherosclerosis. In our previous studies, we have shown that mtDNA mutation are correlated with conventional atherosclerosis and cardiovascular disease risk factors [89].

Foam cell formation in atherosclerosis might be tightly related to mitochondrial dysfunction that impairs FAO and TCA cycles [90]. In that case, mutations in mtDNA genes related to the OXPHOS pathway might disrupt FAO, since it interconnects with TCA. Besides, mtDNA mutations and defective mitophagy may promote proinflammatory response in macrophages and provoke local inflammation in the vascular wall that leads to possible M1 macrophage polarization and subsequent massive lipid accumulation and severe atherosclerotic lesion [88].

## 5. Cholesterol Efflux

Cellular cholesterol homeostasis is also maintained by removing free cholesterol from the cell via a specific mechanism called cholesterol efflux. This mechanism starts with conversion of cholesterol ester to free cholesterol by neutral hydrolase of cholesterol esters (NCEH) [91,92]. Hormone-sensitive lipase (Lipe) and carboxyl esterase 3 (Ces3) can hydrolyze cholesterol ester in addition to NCEH. However, it has been shown [62] that the activity and level of *NCEH* expression are several times higher than Lipe and Ces3, which suggests that NCEH may play a key role in the hydrolysis of cholesterol esters [93]. Regulated cholesterol efflux in macrophages is carried out by aqueous diffusion, passive transport via SR-BI, and active transport via ATP-binding cassette transporter G1 (ABCG1) and A1 (ABCA1) [94,95]. Aqueous diffusion provides the flow of free cholesterol through plasma membrane to HDL (high-density lipoprotein) particles. Cholesterol efflux from macrophages is mediated by ABCA1 to Apo AI, by ABCG1 to HDL particles and by passive transport via SR-B1 to HDL particles [96].

ABCA1 and ABCG1 are localized in the plasma membrane and mediate cholesterol efflux to HDL and apolipoprotein AI [95,97]. However, ABCA1 and ABCG1 are not anchored to the membrane and are able to translocate between the plasma membrane and cholesterol-rich endosomes. LXRα, LXRβ, RXR, and PPARγ, which are transcription factors in many signaling pathways, upregulate the expression of ABC transporters under conditions of excessive cholesterol accumulation [97,98]. In addition, protein kinases PKA, PKC, and JAK2 provide phosphorylation of ABCA1 and ABCG1, which protects ABC transporters from degradation and thereby increases cholesterol efflux [99]. In contrast, PCSK9 downregulates Abca1 gene expression under cellular cholesterol depletion, but makes only a minor effect on expression of ABCG1 and SR-B1 [100]. The ER stress may provoke overexpression of miRNA-33, which also downregulates ABCA1 expression, and, as a consequence, cholesterol efflux [101]. Moreover, ABCA1-dependent cholesterol efflux may be mediated by autophagy that is regulated by inactivation of mTORC1 [102]. In addition, oxidative stress may also induce autophagy by dysregulation of the Keap1 (Kelch ECH associating protein 1) Nrf2 pathway [103]. Nrf2 increases transcription activation of various autophagy associated genes, including p62 gene, which facilitates mitophagy [102].

SR-B1 is a homolog of CD36 [96]. Numerous studies have delivered conflicting results regarding the contribution of SR-B1 to macrophage cholesterol efflux [104]. The activity of SR-B1 can be either higher or lower than the activity of ABCA1, depending on the cholesterol efflux rate and cellular cholesterol level. However, both transporters act together. It has been shown that SR-BI may participate in the regulation of macrophage apoptosis [105]. Cholesterol overload may provoke macrophage death in atherosclerotic lesions. However, the SR-B1 gene deletion in macrophages may lead to apoptosis inhibition under conditions of cholesterol accumulation. The transcription factors PPARγ and LXRα can regulate SR-BI [106]. It was shown that activation of PI3 and Akt signaling pathways by IGF-1 (insulin-like growth factor 1) may inhibit the LXRα expression, which subsequently leads to a decrease of both SR-B1 and ABC transporters abundancy [107]. The expression level of SR-B1, as well as ABCA1 and ABCG1, may be affected by TLR2, which significantly reduces the expression of transporters in macrophages by regulating the NF-κB signaling pathway [108].

## 6. Conclusions

In the current work, we presented the summarized knowledge obtained both by recent studies and classical works on the involvement of cholesterol metabolism in macrophages in atherosclerosis development and progression. Macrophages are specialized cells that can participate in clearance of the intimal space from native and modified LDL. However, they are faced with the difficult task of catabolism of excess lipids, which may interfere with the physiological cellular lipid metabolism. Cholesterol homeostasis in macrophages is a dynamic balance between cholesterol uptake, de novo synthesis, and efflux. These processes are tightly regulated by cellular signaling systems that are also affected by such events as proinflammatory activation, stimulation of phagocytosis, and induction of autophagy. The signaling networks regulating these key events in macrophages are likely to contain specific proteins that can become therapeutic targets for treatment of atherosclerosis. Among them are scavenger receptors that are responsible for lipid uptake and factors regulating their expression, ABCA1 and ABCG1 transporters responsible for cholesterol efflux, and SREBP family proteins involved in lipid biosynthesis. The link between macrophage pro- or anti-inflammatory polarization and alteration of cholesterol metabolism appears to be especially interesting in the context of atherosclerosis. Proinflammatory macrophage polarization is accompanied by decreased lipolysis and enhanced uptake of lipoproteins through an unspecific way leading to foam cell formation, while alternative polarization, induced by IL-4 enhances lipolysis. Despite the substantial amount of data accumulated on lipid homeostasis in macrophages, much work remains to be done to improve our understanding of the mechanistic details of foam cell formation and to translate this knowledge to therapeutic opportunities. The root cause of lipid accumulation by macrophages still remains to be identified: it may be a specific reaction of cells on mLDL or other environmental factors, as well as a response to pro- or anti-inflammatory cytokines or other signal mediators. In any case, the process of excessive lipid accumulation in macrophages is unlikely to be a simple metabolic defect, taking into account upregulation of scavenger receptors that interact with mLDL, and the fact that alternatively polarized M2 macrophages have only marginal lipid accumulation. Signals appearing at the organism level, such as systemic inflammatory condition, can also have an effect on arterial wall cells, including macrophages recruited to the subendothelial space, facilitating lipid accumulation and atherosclerosis development. The exact factors and signaling pathways mediating these effects should be identified by future studies. Another line of future research is the study of mtDNA mutations that are likely to be involved in both cellular lipid metabolism and cellular inflammatory response, but remain insufficiently studied to date to leverage their potential for diagnostic and therapy. Obtaining answers to the questions listed above will improve our understanding of atherosclerosis development and, as a consequence, our ability to find novel and effective therapies.

## Figures and Tables

**Table 1:** Key components of macrophage lipid metabolism.

Process	Main Participants of Processes	Regulation	Cell Compartment
Receptor-mediated LDL uptake	TLR4, MyD88	SYK/PI3K/Akt signaling pathway	Plasma membrane
	LDLR, LRP1	AP-2, CCC, WASH, PCSK9, LXR, SREBP1c, SREBP2	
	SR-AI, SR-AII	Sac1, Sac3, LKB1	
	CD36	Kinases Src, Jnk, Rac (GTPase) protein, NF-κB, CD146, Nrf1	
	LOX-1	PCSK9	
	CD68, SCARF1, SR-PSOX/CXCL16		
Fluid-phase pinocytosis (receptor-independent LDL uptake)		PI3K, LXR, M-CSF receptor, PKC	Plasma membrane
LDL lysis	Lysosomal lipoprotein lipase, lysosomal acid lipase (LAL)	Perilipins, Lipases and Rab GTPases	Lysosomes
Intracellular lipid trafficking	NPC 1 and 2	LAMP 1 and 2, LBPA	Lysosomes
	ORP, VASt, START		Cytoplasm
Free cholesterol esterification	ACAT1	Kinase signaling cascade MAP, Jak, Erk, and signaling cascade Jnk, NF-κB	Endoplasmic reticulum
Fatty acid β-oxidation	Acyl-CoA synthetase, carnitine acyltransferase 1, β-oxidation pathway enzymes	Malonyl-CoA	Cytoplasm, Mitochondria
Fatty acid synthesis	Acetyl-CoA, ACC, FASN, malonyl-CoA, fatty acyl-CoA	SREBP1, USF1, and USF2	Mitochondria, Endoplasmic reticulum, Cytoplasm
Triacylglycerol synthesis	DGAT1, DGAT2, diacylglycerol, fatty acyl-CoA		Endoplasmic reticulum
Lipid droplet formation	ACAT1, ACAT2, DGAT1, DGAT2, FIT1, FIT2, seipin, Pln1, CCTα		Endoplasmic reticulum
Cholesterol synthesis	HMG-CoA reductase, acetyl-CoA, HMG-CoA, Mevalonate	SREBP2, SCAP, INSIG1	Endoplasmic reticulum
Generation of free cholesterol and fatty acids from cholesterol esters	NCEH, Lipe, Ces3		Endoplasmic reticulum
Free cholesterol efflux to HDL	ABCA1, ABCG1, SR-BI	LXRα, LXRβ, RXR, PPARγ, PCSK9, DAPK1, NF-κB, PKA, PKC, JAK2, miRNA-33, mTORC1, Keap1/Nrf2 pathway, IGF-1, PI3, and Akt signaling pathways, TLR2	Plasma membrane
